# Potential molecular targets on which methylene blue acts to promote its effects on cardiac electrophysiology, myocardial injury, and oxidative stress in spontaneously hypertensive rats submitted to cardiac ischemia and reperfusion

**DOI:** 10.1590/acb411826

**Published:** 2026-05-18

**Authors:** Rildo Yamaguti Lima, Tania Carmen Peñaranda Govato, Erisvaldo Amarante de Araújo, Marcelo Pires-Oliveira, Paulo Sérgio de Araujo Sousa, Jefferson Almeida Rocha, Leiz Maria Costa Véras, Sandra Augusta Gordinho Pinto, Afonso Caricati-Neto, Fernando Sabia Tallo, Célia Maria Camelo Silva, Francisco Sandro Menezes-Rodrigues

**Affiliations:** 1 Universidade Federal de São Paulo – Postgraduate Program in Cardiology – São Paulo (SP), Brazil.; 2 Centro Universitário UNIME – Medical Course – Department of Medicine – Lauro de Freitas (BA), Brazil.; 3 Universidade Federal do Delta do Parnaíba – Research Center on Biodiversity and Biotechnology – Parnaíba (PI), Brazil.; 4 Universidade Federal do Maranhão – São Bernardo (MA), Brazil.; 5 Universidade Federal de São Paulo – Department of Pharmacology – São Paulo (SP), Brazil.; 6 Universidade Federal de São Paulo – Postgraduate Program in Interdisciplinary Surgical Science – São Paulo (SP), Brazil.

**Keywords:** Ischemia, Reperfusion, Methylene Blue, Rats, Inbred SHR, Wounds and Injuries, Vasoplegia

## Abstract

**Purpose:**

Although methylene blue (MB) has long been used to treat vasoplegic syndrome, its pharmacodynamics and cardiovascular effects remain unclear. Therefore, we decided to evaluate the potential molecular targets of MB through molecular docking and its cardioprotective and antioxidant actions.

**Methods:**

Adult male spontaneously hypertensive rats (SHR) were allocated into four groups: sham-operated; treated with vehicle and subjected to cardiac ischemia and reperfusion (CIR) (SS+CIR); treated with 2 mg/kg of MB before cardiac ischemia and reperfusion (MB+ISQ); and treated with 2 mg/kg of MB before cardiac reperfusion (ISQ+MB). The animals were monitored with electrocardiogram to assess the incidence of ventricular arrhythmias (VA), atrioventricular blocks (AVB), and lethality (LET). Blood samples and hearts were collected for histopathological analysis, serum creatine kinase MB dosage, and measurement of lipid hydroperoxide (LH) in myocardium serum creatine kinase MB.

**Results:**

The incidences of AVB and LET, serum CK-MB concentration, and myocardial injury increased in the MB+ISQ group, but not in the ISQ+MB group when compared with the SS+CIR group. LH concentration decreased in both MB-treated groups when compared with the SS+CIR group.

**Conclusion:**

These results indicate that MB produces a cardiotoxic effect only when administered before CIR, but not when administered before cardiac reperfusion.

## Introduction

Cardiovascular diseases (CVD) are the biggest cause of death worldwide, and ischemic heart diseases (IHD), including hypertension and acute myocardial infarctions, are one of the leading causes of death in both developed and undeveloped countries^1-3^. Although the main form of treatment for IHD is recovery of coronary blood flow post ischemia, cardiac reperfusion can also lead to oxidative stress and electrochemical imbalances in cardiomyocytes, as well as severe cardiac arrythmias^4,5^.

Intense inflammation, with increased pro-inflammatory cytokines, such interleukins-1 and -6, and tumoral necrosis factor-α, and oxidative stress mostly from nitric oxide (NO) metabolism are the main causes of these reperfusion injuries^6,7^. These lead to increased adrenal secretion of catecholamines, vasoconstriction and blood pressure. However, this overproduction eventually results in catecholamine depletion and intense and continuous relaxation of vascular smooth muscle because of the desensitization of α1-adrenoceptors found in blood vessels. This condition, the vasoplegic syndrome (VS), is characterized by excessive vasodilation when the impact of NO is predominant and there is no sympathetic equivalent^6-11^.

A popular model for essential hypertension, cardiac hypertrophy, and, crucially, autonomic imbalance is the spontaneously hypertensive rat (SHR). Hypertension is one of the main risk factors for heart failure and IHD, but few experimental investigations have examined cardioprotective treatments that increase parasympathetic nervous system activity in hypertension models, despite the essential role of autonomic dysfunction apparent connection. Multiple vasopressors, such as catecholamine-sparing agents—the most often utilized being norepinephrine and vasopressin—, may be required in this situation in order to accomplish and sustain the hemodynamic target^12,13^. Vasodilation and a reduction in catecholamine activity result from reactive oxygen species (ROS)-induced soluble guanylate cyclase (sGC) in vascular smooth muscle producing too much NO^3,7^. Because it affects NO synthase and sGC activity, methylene blue (MB) is an alternative adjuvant option^3,7^. Over the past ten years, MB has been used to treat anaphylaxis, septic shock, and vasoplegia in heart surgery. Without affecting mortality, MB usage has been linked globally to elevated average blood pressure and systemic vascular resistance^14-16^.

MB is the primary treatment used to raise systemic vascular resistance and lower vasopressor needs in human VS^14-16^. Several studies have suggested that the vascular effects of MB were initially attributed to the pharmacological blockade of guanylate cyclase (GC) and attenuation of oxidative stress^14-16^, and the cellular and molecular mechanisms involved in these effects remain under investigation. Thus, aiming to contribute to the advancement of this knowledge, we decided with the present study to investigate the potential molecular targets on which MB acts to promote its effects on cardiac electrophysiology, myocardial injury and oxidative stress in SHR submitted to cardiac ischemia and reperfusion (CIR).

## Methods

### Animals

Adult male SHR were obtained from the Center for the Development of Experimental Models in Medicine and Biology at the Universidade Federal de São Paulo (UNIFESP) and housed in standard settings for diet, hydration, and environment. All experimental procedures complied with the Brazilian National Council for the Control of Animal Experimentation guidelines, as well as National Institutes of Health’s Guide for the Care and Use of Laboratory Animals, and were authorized by UNIFESP’s Ethics Committee on Animal Use (9447210317, approved in 2017).

#### Molecular docking

The threedimensional (3D) structures of the proteins were obtained from the Protein Data Bank with the codes 1CFF, 1GOS, 4D10, 6JT2 and 6OIK. All molecular docking procedures were performed using the Autodock 4.2 package. Proteins and ligands were prepared for molecular interaction simulations with AutoDock Vina. Partial Gasteiger charges were calculated after addition of polar hydrogens, and non-polar hydrogen atoms from the protein and ligand were subsequently added. A cubic box of 30×30×30 points was generated at the active site of the protein to delineate the interaction site of the ligand with the protein. Other docking parameters have been set to default values. The resulting docked conformations were grouped into families according to the root-mean-square deviation. For a more detailed analysis, the coordinates of the selected complexes were chosen based on the best binding energies and combined with visual inspection^17-20^.

### Surgical protocol for induction of cardiac ischemia and reperfusion

The surgical procedures to induce CIR and electrocardiogram data acquisition were performed as previously described^21,22^. After 10 minutes of ischemia of the left anterior descending artery, we allowed for 75 minutes of coronary reperfusion.

SHR were randomly allocated into four experimental groups:

- Sham group (n = 10): underwent all surgical procedures, except for the garroting of the left anterior descending coronary artery;
- SS+CIR group (n = 14): received 0.9% saline solution (SS) via the left femoral vein prior to ischemia;
- MB+ISQ group (n = 14): received 2 mg/kg of MB via the left femoral vein prior to ischemia;
- ISQ+MB group (n = 14): received 2 mg/kg of MB via the left femoral vein after ischemia, but before reperfusion.

### Biochemical evaluation of serum markers of cardiac injury

Blood samples were extracted from the abdominal aorta at the end of the reperfusion period, centrifuged for 40 minutes at 2,500 rpm, 5°C, and stored at -20°C. Using a commercial kit (Katal Biotecnológica Indústria e Comércio, Belo Horizonte, MG, Brazil) and a kinetic ultraviolet approach with a measuring point of 340 nm, creatine kinase MB fraction (CK-MB) was quantitatively determined^3^.

### Biochemical analysis of cardiac lipid hydroperoxides

Samples of rat heart tissue were homogenized, and centrifuged for 10 min at 10,000 rpm, at 20°C. The supernatant was then gathered for measurement of total protein and lipid hydroperoxides (LH)^3^, using a technique based on hydroperoxide oxidation of Fe^2+^ into Fe^3+^, as previously described.

### Histopathological analysis of left ventricle myocardial tissue

Cardiac left ventricle fragments were divided into three portions (apex, mid, and distal regions) with roughly comparable thickness using axial cross-sections. Mid sections were cross-sectioned (4–5 µm width) and stained with hematoxylin-eosin (HE). A qualified pathologist blindly assessed the slides using a light microscopy (×400 and ×1,000) for lesions associated with CIR: presence of hyperemic blood vessels, pyknosis, inflammatory infiltration, cardiomyocyte degeneration, loss of striation, and interstitial edema^3^.

### Data collection to carry out molecular docking

#### Methylene blue design and optimization

The three-dimensional (3D) structure of MB was designed and optimized using GaussView 5 and Gaussian 09w software, respectively. Optimization was performed using the density functional theory method with the B3LYP hybrid functional and the 6-31G(d) basis set^17^.

### Statistical analysis

Fisher’s exact test was used to compare ventricular arrhythmias (VA), atrioventricular blocks (AVB), and lethality (LET) incidences. CK-MB and LH levels were expressed as mean ± standard error of the mean (SEM) and compared with one-way analysis of variance (ANOVA) with Tukey’s post hoc test. Statistical significance was defined as a *p*-value ≤ 0.05. GraphPad Prism 8.0 (GraphPad Software Inc., La Jolla, CA, United States of America) was used for statistical analysis^7^.

## Results

### Methilene blue molecular docking × molecular targets in the cardiomyocyte

The results regarding the molecular coupling of MB with proteins are presented in Table 1. The optimal binding energy parameters were obtained from the interaction between MB and 1GOS, in the second active site. Affinity was observed with binding energy equal to -8.9 kcal.mol^-1^ (Table 1). In the formation of this complex, it was possible to observe interactions with the amino acids Ile B: 198, Phe B: 168, Tyr B: 435, Cys B: 397, Gly B: 58, Met B: 436, Gly B: 434, Ser B: 59, Tyr B: 398, Tyr B: 60, Phe B: 343, Gln B: 206, Tyr B: 326, Leu B: 171, Ile B: 199, and Cys B: 172 of the active site of the protein with MB.

**Table 1:** Molecular affinity parameters of methylene blue with proteins 1CFF, 1GOS, 4D10, 6JT2, and 6OIK.

Complex (ligand-protein)	ΔG bindum (kcal . mol^-1^)	Interactions of ligands with b protein residues*
Methylene blue-1CFF	-6	Tyr A: 99, Asn A: 137, Glu A: 139, Thr A: 146, Val A: 142, Glu A: 83, Tyr A: 138, Ser A: 81 e Glu A: 82
Methylene blue-1GOS (first active site)	-8.6	Met A: 436, Gly A: 434, Tyr A: 398, Tyr A: 435, Gln A: 206, Cys A: 172, Ile A: 198, Leu A: 171, Tyr A: 326, Ile A: 199, Phe A: 343, Tyr A: 60, Cys A: 397, Gly A: 58 e Arg A: 42
Methylene blue- 1GOS (second active site)	-8.9	Ile B: 198, Phe B: 168, Tyr B: 435, Cys B: 397, Gly B: 58, Met B: 436, Gly B: 434, Ser B: 59, Tyr B: 398, Tyr B: 60, Phe B: 343, Gln B: 206, Tyr B: 326, Leu B: 171, Ile B: 199 e Cys B: 172
Methylene blue-4D10 (first active site)	-7.1	Glu E: 202, Ala E: 223, Ser E: 201, Lys E: 219, Cys E: 218, Tyr E: 221, Phe E: 214, Cys E: 145, Trp E: 146, Phe E: 186, Gln E: 204, Gly E: 184 e Leu E: 183
Methylene blue-4D10 (second active site)	-7	Ala M: 223, Glu M: 202, Ser M: 201, Lys M: 219, Cys M: 218, Tyr M: 221, Phe M: 214, Cys M: 145, Trp M: 146, Phe M: 186, Gln M: 204, Gly M: 184 e Leu M: 183
Methylene blue-6JT2	-6.9	Lys B: 478, Cys A: 533, Glu A: 526, Arg A: 593, Val A: 525, Ile A: 528, Cys A: 595, Gly B: 476, Val B: 475, Val A: 601, Thr A: 602, Asp B: 477, Phe B: 430, Asn A: 605 e Asn B: 431
Methylene blue-6OIK	-8	Phe R: 181, Asn R: 410, Trp R: 422, Asn R: 419, Thr R: 423, Thr R: 84, Tyr R: 80, Tyr R: 83, Ile R: 178 e Tyr R: 177

* Obtained with BIOVIA Discovery Studio Visualizer.

Source: Elaborated by the authors.

The molecular docking of MB carried out in the present study presented possible pharmacological molecular targets with links that varied between excellent and good, among them, monoamine oxidase-B (MAO-B), Sinalosoma COP9, enzyme GC, muscarinic acetylcholine receptor type M_2_ (MR_2_) and calmodulin CaM, with some of them even presenting more than one active binding site for MB, in this case MAO-B and the Sinalosome COP9, indicating that the response of the cardiac muscle to CIR under the effects of MB could be mediated not only by the modulation of NO through the inhibition of GC, as studies in this regard describe.

### Ventricular arrhythmias, atrioventricular blocks, and lethality incidence

**Figure 1:**
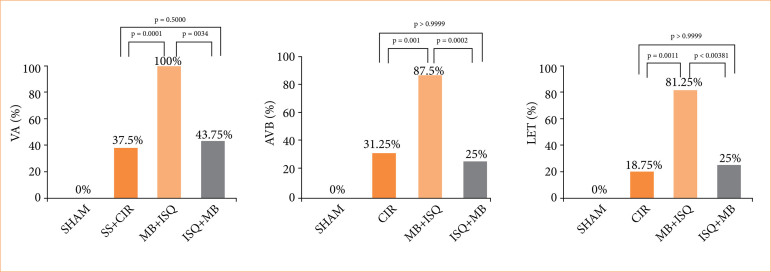
The prevalence of ventricular arrhythmias (VAs), atrioventricular block (AVB), and lethality (LET) in the ISQ+MB, MB+ISQ, SS+CIR, and sham groups. Compared to the SS+CIR group, the MB+ISQ group showed a significant increase in VA, AVB, and LET. Comparing ISQ+MB, which was given after ischemia but before reperfusion, to SS+CIR, revealed a significant decrease in VA, AVB, and LET. VA, AVB, and LET were absent in sham rats. Fisher’s exact test was utilized to compare the data.

ISQ: ischemia; MB: methylene blue; SS: saline solution; CIR: cardiac ischemia and reperfusion. Source: Elaborated by the authors.

When compared to the SS+CIR (control) and sham groups, Fig. 1 illustrates that the incidence of VA, AVB, and LET was negatively impacted by the administration of MB either prior to ischemia or prior to reperfusion. In comparison to SS+CIR, MB significantly enhanced the incidence of VA (100%), AVB (100%), and LET (100%) when given prior to myocardial ischemia (MB+ISQ) (VA = 85.7%; AVB = 71.4%; LET = 64.2%). As opposed to SS+CIR, MB shown a considerably protective effect and decreased the incidence of VA (42.8%), AVB (8.5%), and LET (21.4%) when given after myocardial ischemia but before to reperfusion (ISQ+MB).

### Results obtained through histopathological analysis of myocardium from spontaneously hypertensive rat groups

**Figure 2:**
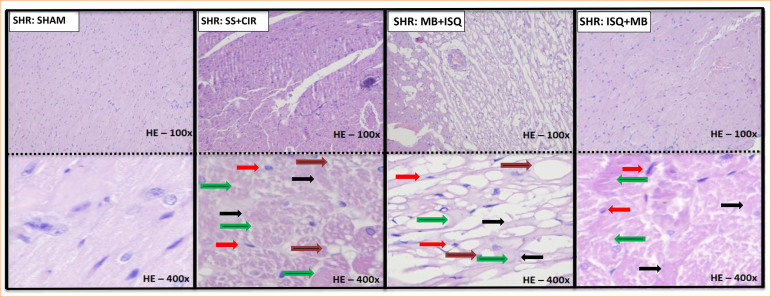
Histopathological examination of the heart tissue of rats from the experimental groups sham, SS+CIR, MB+ISQ, and ISQ+MB is shown in representative photomicrographs. The myocardium in the sham group is distinguished by striated cells with well-centralized nuclei of normal size and color, as well as the lack of necrosis. The SS+CIR group has severe coagulation-induced necrosis, characterized by pyknotic cells (decentralized nuclei and chromatin condensation), a significant proportion of cells experiencing karyolysis (nucleus absence and cytoplasmic eosinophilia), swelling, and vacuolization. Like this, the MB+ISQ group exhibits severe coagulation-induced necrosis, severe tissue loss, myocytolysis, muscle fiber swelling (a reversible increase in cell volume brought on by water accumulation, ionic imbalance), vacuolation, and cells with reduced nuclei or karyolysis. In contrast, the myocardium in the MB+ISQ group exhibits modest coagulation-induced necrosis, along with mild karyolysis, swelling, and the lack of vacuolization, yet a significant portion of the striated cells (hematoxylin and eosin at 400× magnification) are preserved.

ISQ: ischemia; MB: methylene blue; SS: saline solution; CIR: cardiac ischemia and reperfusion; SHR: spontaneously hypertensive rats. Source: Elaborated by the authors.

When compared to SS+CIR (control), Fig. 2 demonstrates that the histopathological effects of MB treatment prior to either ischemia or reperfusion on the cardiac tissue were opposite. The cardiac tissue in the sham group has striated cells with well-centralized nuclei that are normal in size and color, and there is no necrosis present. The cardiac tissue in the SS+CIR group exhibits severe coagulation-induced necrosis, with pyknotic cells and a significant number of cells undergoing karyolysis, swelling, and vacuolization. Similar results are shown in the MB+ISQ group, including coagulation-induced necrosis, severe tissue loss, myocytolysis-affected areas, swollen, vacuolated muscle fibers, and cells with decreased nuclei or karyolysis. However, in the ISQ+MB group, the coagulation-induced necrosis is less severe, exhibiting minimal pyknosis, mild karyolysis, swelling, and no vacuolization, while a significant portion of the striated cells are preserved.

### Effects of methylene blue on the serum concentration of creatine kinase-MB fraction in spontaneously hypertensive rat groups

**Figure 3:**
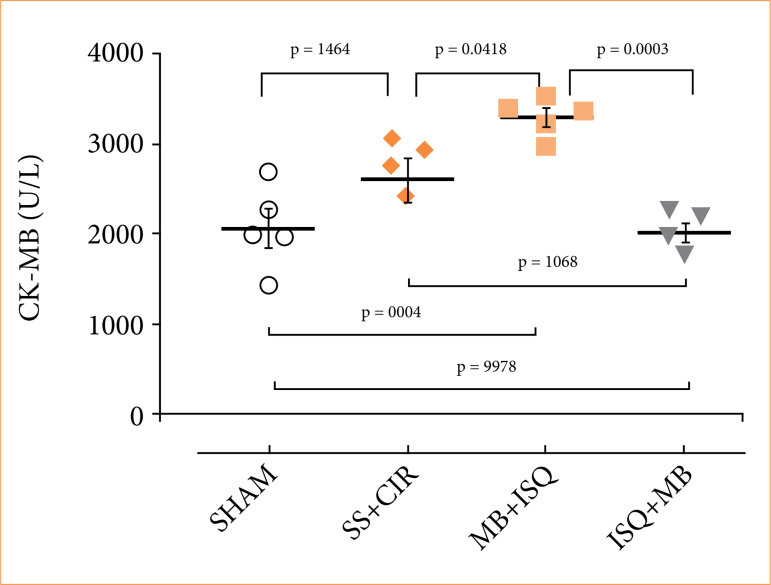
Serum concentrations of the myocardial injury marker CK-MB (U/L). Treatment with MB before ISQ increased the serum concentration of CK-MB when compared to the other groups. Analysis of variance and Tukey’s post hoc test were used to compare the data, which are displayed as scatterplots with the mean ± standard error of the mean.

ISQ: ischemia; MB: methylene blue; SS: saline solution; CIR: cardiac ischemia and reperfusion; CK-MB: creatine kinase-MB. Source: Elaborated by the authors.

The MB fraction of the enzyme creatine kinase is a marker of myocardial injury, used in clinical cardiology for the diagnosis of acute myocardial infarction. No differences were observed between the serum CK-MB concentrations of the sham (2,068 ± 208.1 U/L), SS+CIR (2,606 ± 228.1 U/L) and ISQ+MB (2,025 ± 86.7 U/L) groups. However, the serum CK-MB concentration of the MB+ISQ (3,306 ± 95.1 U/L) group was higher than the concentrations of the other groups. These results demonstrate that treatment of the animals with MB before cardiac ischemia promoted a cardiotoxic effect (Fig. 3).

### Lipid hydroperoxide concentration in the heart

**Figure 4:**
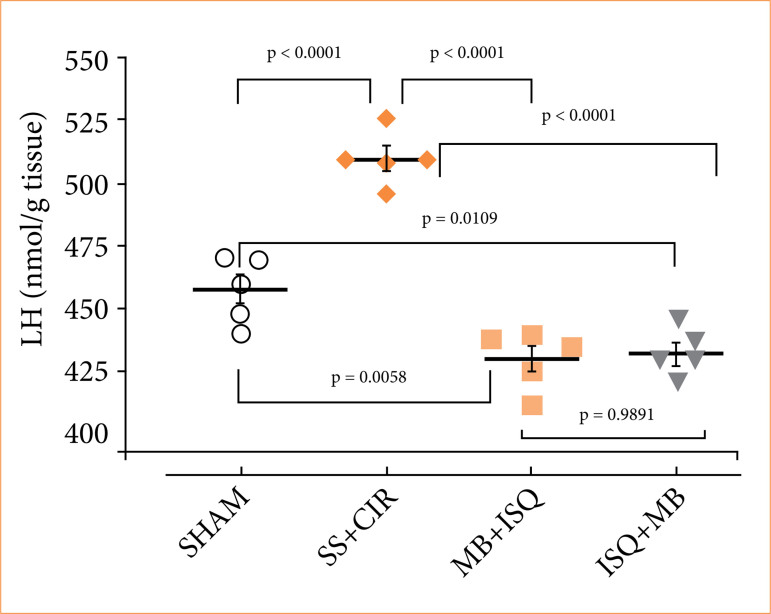
The amounts of LH, a biochemical indicator of oxidative stress, in the heart of each experimental group. Treatment with MB before ISQ and before cardiac reperfusion decreased the cardiac concentration of LH when compared to the SS+CIR and sham groups. Analysis of variance and Tukey’s post hoc test were used to compare the results, which are presented as scatterplots with the mean ± SEM (n = 5).

ISQ: ischemia; MB: methylene blue; SS: saline solution; CIR: cardiac ischemia and reperfusion; LH: lipid hydroperoxide. Source: Elaborated by the authors.

Cardiac tissue from the MB+ISQ, ISQ+MB, and sham groups had substantially lower LH concentrations (nmol/g tissue) than the SS+CIR group, as seen in Fig. 4. When compared to the SS+CIR group, MB treatment before or during ischemia significantly decreased cardiac LH, just as MB treatment before or during ischemia also significantly decreased cardiac LH when compared to the sham group.

## Discussion

MB has been used for the treatment of several CVD, including cardiovascular disorders related to septic shock and VS. One of the main causes of severe complications and early postoperative death following cardiopulmonary bypass is the VS^20^. Between 5 and 25% of people having heart surgery will develop VS. The first-line vasopressor medication for VS is now thought to be norepinephrine. Commonly used, catecholamines in heart surgery can exacerbate pulmonary hypertension (PH), and medications that lower PH, like prostaglandins, phosphodiesterase inhibitors, and NO, can exacerbate vasoplegia following cardiopulmonary bypass^20-29^.

VS is a serious and deadly complication that affects patients undergoing cardiovascular surgical procedures using extracorporeal circulation^22^. Patients with VS present cardiovascular complications that result in an increased rate of morbidity and mortality, such as hypotension, normal or high cardiac output, low resistance systemic vascular disease, and decreased filling pressures^20-29^. Under these conditions, MB has been indicated to increase systemic vascular resistance and reduce doses of vasopressor medications used in the drug treatment of VS^3,7^. Although the cardiovascular effects of MB were initially attributed to the pharmacological blockade of GC, and additionally to the reduction in the generation of highly reactive chemical species or free radicals^30,31^, cellular and molecular mechanisms involved in these effects remain under investigation. Aiming to advance knowledge of the spectrum of action of MB, the cardiovascular actions and effects of MB in an animal model of primary hypertension submitted to CIR were investigated in the present study.

The first step was to verify possible pharmacological targets in cardiac tissue, beyond speculation about their effects being related only to the modulation of NO activity. Monoamine oxidase-B (MAO-B), Sinalosoma COP9, enzyme GC, muscarinic acetylcholine receptor type M2 (MR2), and calmodulin CaM were among the potential pharmacological molecular targets with links ranging from excellent to good, according to the molecular docking of MB conducted in this study. Some of these even presented multiple active binding sites for MB, in this case MAO-B and the Sinalosome COP9, suggesting that the response of the cardiac muscle to CIR under the effects of MB could be mediated not only by the modulation of NO through the inhibition of GC, as studies in this regard describe.

MB is known as a MAO inhibitory agent. Considering this inhibitory action and its cardiac repercussion on CIR events, MAO are proteins located in the outer mitochondrial membrane and use catecholamines as substrates. More specifically, MAO-B is expressed in the heart and preferentially oxidizes β-phenylethylamine, dopamine and histamine. Some studies have demonstrated that MAO-B activity in the heart muscle, under stressful situations such as CIR, causes structural and functional disorders^32-34^. Although they use different substrates, MAO-A and -B catalyze the same oxidative deamination reaction, which results in the formation of aldehyde, ammonia and H_2_O_2_. Both the aldehyde 3,4-dehydroxyphenylacetaldehyde (DOPAL), resulting from the enzymatic action on dopamine, and H_2_O_2_ are extremely toxic substances, which increase oxidative stress, the destruction of cellular structures, mainly contributing to mitochondrial dysfunction and cell death. Therefore, the inhibition of MAO-B activity in CIR, in addition to reducing the speed of neurotransmitter degradation, increasing their bioavailability, provides a reduction in diastolic pressure in these events, in which there are hypercontraction of myocytes and increase in diastolic pressure. This inhibition would also be directly related to the reduction in ROS production and mitochondrial dysfunction, due to the reduction in the production of H_2_O_2_, and aldehydes, oxidative stress, and LP trigger the toxic accumulation of reactive aldehydes, such as 4-HNE and malondialdehyde, which inactivate a series of macromolecules, including the proteasome and the electron transport chain in mitochondria^32-34^.

We found in this study that calmodulin (CaM) also constitutes one of the molecular targets of MB. CaM is a multifunctional Ca^2+^-dependent kinase enzyme with diverse functions in cardiomyocytes, mainly the phosphorylation of proteins involved in the regulation of intracellular Ca^2+^ ion concentrations, including relevant ion channels, such as SERCA, RyR, phospholamban (PLB) and channel modulation of L-type Ca^2+^ (CCVD) and other components of the AECC system, in addition to being responsible for the activation of NOS of endothelial origin (eNOS), which synthesizes NO from L-arginine^35,36^.

With the possible inhibitory effect of this enzyme by MB, there would be a reduction in eNOS activity and consequently in NO biosynthesis with important implications for CIR, described later. Regarding the action on the modulation of [Ca^2+^]c, it is possible to infer that the inhibition of CaM activity in RyR could reduce the release of this ion from the RES to the cytosol, thus reducing its cytosolic concentration. In the same way, the inhibition of CaM that phosphorylates PLB, which in turn reduces the activity of SERCA, resulted in an increase in the activity of the latter, thus increasing the reuptake of Ca^2+^ by the RES. On the other hand, still in relation to intracellular concentrations of Ca^2+^, inhibition of CaM would function as a factor in increasing the entry of this ion from the extra medium into the intracellular environment, as it would result in the inactivation of the negative sensor calcium dependent inactivation (CDI) of the CCVD^35-37^.

Under the conditions already mentioned, the inhibition of CaM by MB becomes more favorable to the regular functioning of cardiomyocytes during CR, as the resulting reduction in NO production is more relevant during this period, in which the abrupt introduction of O_2_ occurs in an anaerobic environment. and the consequences of the NO reaction with O_2_, as well as the inactivation of the CDI sensor of type L CCVD would considerably increase intracellular Ca^2+^ concentrations and would also hinder the repolarization of cardiomyocytes, even with the increase in SERCA and RYR activities^6,38^.

Studies using M_2_-selective muscarinic antagonists in cardiomyocytes have shown that M_2_-type muscarinic cholinoceptors mediate vagal negative inotropic and chronotropic effects, therefore slowing atrioventricular node conduction^39-41^. The pharmacological blockade of M_2_ by MB could explain the fact that this medication is not able to reduce the incidence of VA, but still significantly prevents severe post-CIR AVB and LET. The increased incidence of AVB observed mainly during reperfusion has been shown to precede most deaths in the animal model of CIR^3,6,7^. Therefore, treatment with MB before ischemia missed a crucial window to avoid significant delay in AVB and LET and is not more effective than treatment with SS. On the other hand, no significant changes were detected in the serum concentration of CK-MB, reinforcing the idea that MB modulates cardiac electrical activity and AECC, preserving the cardiac structure, and thus producing a cardioprotective effect^7,42^.

Regarding the COP9 Sinalosoma, comprising eight subunits known as CSN1-CSN8, the constitutive photomorphogenesis 9 (COP9) signalosome (CSN) is a multi-protein complex that has been preserved throughout history. CSN’s primary biochemical role is to regulate cullin-RING E3-ligase (CRL) activity by deNEDDylating cullins, which controls protein degradation via the ubiquitin-proteasome system^43^. However, the CSN also acts as a docking platform for signaling proteins. CSN5 carries out the complex’s catalytic deNEDDylase (isopeptidase) activity, but it only works effectively inside the complex’s 3D architectural framework^43^. In addition, the CSN has been linked to a number of human diseases because of its location in a central cellular pathway that is linked to cell responses like cell-cycle, proliferation, and signaling^43^. Through a mechanism separate from NO signaling, Bhansali and Shemshedini^44^ showed that the soluble guanylyl cyclase α1 (sGCα1) is a direct target of the androgen receptor and necessary for the growth of prostate cancer cells. Data demonstrated a new sGCα1 interaction partner in the COP9 signalosome subunit 4 (CSN4). Crucially, sGCα1 proteasomal degradation is inhibited by the CSN4-sGCα1 connection. In line with this, CSN4 disruption resulted in a significant reduction in prostate cancer cell proliferation, which was partially but not entirely restored by sGCα1 overexpression, suggesting that CSN4 may have another target^44^.

Most of the evidence points to a role in cancer, but new data also points to a role for the CSN in cardiovascular and inflammatory disorders. This is because of its function in regulating CRLs, complex-independent interactions of subunits like CSN5 with inflammatory proteins, and components of important inflammatory pathways including nuclear factor kappa-light-chain-enhancer of activated B cells (NF-κB) signaling to proteotoxicity and necrosis in cardiovascular conditions such heart failure and atherosclerosis^44^.

Last but not least, MB binding in GC, perhaps the most discussed mechanism regarding the effects of MB in CIR injuries, essential for the NO signaling pathway, the soluble GC enzyme catalyzes the formation of cGMP from GTP, and the NO/GC/cGMP pathway is involved in the regulation of a wide variety of biological and physiological processes in the mammalian organism^21-29^. GC activated by NO catalyzes the formation of cGMP, this second messenger being directly related to the dephosphorylation of the myosin light chain, inhibition of Ca^2+^ influx, activation of protein kinases, stimulation of membrane Ca^2+^-ATPase and opening of K^+^ channels, playing therefore a fundamental role in cellular signaling and functioning, both in physiological and pathophysiological conditions, such as CIR. In addition to the consequent contributions to ionic dysregulation and contractile machinery previously reported, inhibition of GC can increase the bioavailability of NO for reactions formation of free radicals and increased oxidative stress^3,7,30^.

NO is a soluble gas with an extremely fast half-life (< 10 seconds), produced mainly from the substrate L-arginine by the action of NOS and can have diverse actions, both paracrine and autocrine and endocrine, important in vasodilation, in the inflammatory response, and also as a neurotransmitter in the central nervous system. On the other hand, the presence of unpaired electrons (free radical) makes it a highly reactive chemical agent and, therefore, highly toxic^45,46^. In the presence of O_2_ and superoxide radicals, NO reacts to form other products that are extremely cytotoxic and have a high destructive potential for cellular components, such as lipids, proteins and nucleic acids, including peroxynitrite (ONOO-), in addition to peroxynitrous acid and its decomposition products (HO- and NO_2_), highly oxidizing substances^45,47,48^.

One of the biggest problems with the exacerbated formation of ROS is LP, since due to physicochemical characteristics, polyunsaturated fatty acids are the main targets of these substances, giving rise to highly toxic products, such as malondialdehyde and 4-hydroxynonenal, which can increase the permeability of the inner mitochondrial membrane, alter membrane fluidity, transport electrons and produce H_2_O_2_, in addition to promoting the oxidation of pyridine nucleotides, which impairs membrane permeability, uncoupling phosphorylation oxidative activity and adenosine triphosphate (ATP) production in mitochondria^47-50^.

Although the generation of ROS during heart failure (HF) is an important factor in the occurrence of cardiac injuries resulting from CIR, no important differences were observed in relation to the serum concentration of the cardiac injury markers studied in SHR. According to the literature, endothelial dysfunction associated with human primary hypertension and in SHR causes a reduction in the bioavailability of NO, which plays a significant role in the pathogenesis of hypertension. This fact not only decreases the vasodilatory effect of NO, but also reduces the stimulation of the expression of vascular growth factors, such as vascular endothelial growth factor (VEGF)^13,51,52^. Thus, it is plausible that MB administration before CIR can dramatically reduce the natural cardioprotection against CIR present in SHR^6^. The most relevant effect of MB during CIR, especially in SHR animals, may not be related to blocking eNOS activity, but other effects, for example, MAO blockade that interferes with cardiac autonomic balance. This could contribute to the explanation of the results observed in SHR animals.

Thus, the biggest problem during CIR is centered on the role of the progressive increase in [Ca^2+^]c, resulting from the abrupt interruption of molecular oxygen, fundamental for cellular bioenergetics, mainly at mitochondrial levels, culminating in ionic imbalance and membrane degradation, which has this condition worsened by the activity of MB, if on one hand the inhibitory activity of MB to MAO-B and inactivation of CaM with consequent inactivation of proteins modulated by CaM such as eNOS can favor the reduction or delay of injury and death cellular, reducing the production of NO and the degradation of neurotransmitters that would result in the formation of aldehydes and H_2_O_2_.

A reduction that provides greater bioavailability of MAO substrate neurotransmitters, favoring the reduction of diastolic pressure, seems to overlap with these other negative factors, inhibition of CaM that phosphorylate ionic transporters, mainly the CCVD of the sarcolemma, cause the inactivation of the CDI, favoring an even more accentuated increase in Ca^2+^ into the cell, increasing the reuptake of this ion by SERCA and decreasing the activity of RyR, another factor of great importance. importance is the blockade of GC activity exerted by MB, an enzyme that catalyzes the production

of cGMP, a second messenger whose activity results in the dephosphorylation of myosin and activation of Ca^2+^ extrusion mechanisms, thus it is possible to infer that the cardiotoxic activity of MB together. Possible cardioprotective activities overlap with their molecular targets during HF, favoring cardiac injuries, excitation-contraction uncoupling, and the worsening of arrhythmias.

## Conclusion

Our results demonstrated that the administration of MB before CIR promoted an increase in arrhythmias and LET, increased cardiac injury and myocardial injury when administered before CIR. However, such effects were not observed when MB was administered before RC.

## Data Availability

Data sharing is not applicable.
